# Identification of Health Effects of Complex Air Pollution in China

**DOI:** 10.3390/ijerph191912652

**Published:** 2022-10-03

**Authors:** Yuxin Zhao, Xingqin An, Zhaobin Sun, Yi Li, Qing Hou

**Affiliations:** 1 School of Atmospheric Physics, Nanjing University of Information Science & Technology, Nanjing 210044, China; 2 State Key Laboratory of Severe Weather of CMA, Chinese Academy of Meteorological Sciences, Beijing 100081, China; 3 Institute of Urban Meteorology, China Meteorological Administration, Beijing 100089, China

**Keywords:** complex air pollution, mortality, generalized additive model, pollution types

## Abstract

After the Chinese government introduced a series of policies to strengthen the control of air pollution, the concentration of particulate matter has decreased, but the concentration of ozone has increased, and the problem of complex air pollution still exists, posing a serious threat to public health. Therefore, disentangling the health effect of multi-pollutants has been a long-discussed challenge in China. To evaluate the adverse effects of complex air pollution, a generalized additive model was used to assess the health risks of different pollution types in eight metropolises in different climates in China from 2013 to 2016. Instead of directly introducing multiple pollutant concentrations, we integrated the concentration levels of PM_2.5_, NO_2_, and O_3_ into a set of predictors by grouping methods and divided air pollution into three high single-pollutant types and four high multi-pollutant types to calculate mortality risk in different types. The comprehensive results showed that the impact of high multi-pollutant types on mortality risk was greater than that of high single-pollutant types. Throughout the study period, the high multi-pollutant type with high PM_2.5_, NO_2_, and O_3_ and the high multi-pollutant type with high PM_2.5_ and NO_2_ were more associated with death, and the highest RRs were 1.129 (1.080, 1.181) and 1.089 (1.066, 1.113), respectively. In addition, the pollution types that most threaten people are different in different cities. These differences may be related to different pollution conditions, pollutant composition, and indoor–outdoor activity patterns in different cities. Seasonally, the risk of complex air pollution is greater in most cities in the warm season than in the cold season. This may be caused by the modifying effects of high temperature on pollutants in addition to different indoor–outdoor activity patterns in different seasons. The results also show that calculating the effect of individual air pollutants separately and adding them together may lead to an overestimation of the combined effect. It further highlights the urgency and need for air pollution health research to move towards a multi-pollutant approach that considers air pollution as a whole in the context of atmospheric abatement and global warming.

## 1. Introduction

Nowadays, air pollution is one of the major health issues facing the world’s metropolitans, especially in developing countries [1,2,3,4,5]. As the largest developing country in the world, China has a large population, higher energy consumption and more serious pollution problems.

Although the Chinese government has issued a series of policies to step up the fight against air pollution, the situation remains grim. On the one hand, most of the policies, such as the Action Plan for The Prevention and Control of Air Pollution mainly aim at the emission reduction policy of inhalable particulate matter. As the emission of ozone and other pollutants has not been effectively limited, the problem of complex air pollution is prominent, especially in key cities and regions. On the other hand, the World Health Organization (WHO) issued the latest revised Global Air Quality Guidelines [6], tightened the annual average target value of PM_2.5_, PM_10_, NO_2_, and other long-term exposure indicators based on new evidence of the health effects of low concentration levels and long-term exposure to pollutants. By 2020, although the overall annual average concentration of PM_2.5_ was reduced to 33 μg/m^3^ for the first time, 34% lower than in 2015 [7], there was still a big gap between the new AQG target value of 5 μg/m^3^ [6]. Therefore, the problem of complex air pollution is not only prominent now but also continues to exist at least for a while. Under the background of atmospheric emission reduction and global climate change, it is an important challenge to strengthen the coordinated control and treatment of multiple pollutants and effectively solve the regional and complex pollution problems represented by PM_2.5_ and ozone, so as to protect public health. At the same time, it also puts forward higher requirements for research in the field of air pollution and health.

Previous research on air pollution and health has focused on the health effects of individual pollutants. Many studies [8,9,10] have been conducted to ascertain the effects of air pollution on mortality by single-pollutant models, which assess the health effect of one pollutant, primarily the effect of particulate matter (PM) [11,12] (e.g., particulate matter < 10 µm in aerodynamic diameter (PM_10_) and particulate matter < 2.5 µm (PM_2.5_)) and gaseous pollutants [13,14,15] (e.g., nitrogen dioxide (NO_2_), sulfur dioxide (SO_2_), and ozone (O_3_)) on health, especially mortality outcomes. However, complex air pollution exists as a complex mixture whose nature and consequences are without doubt multi-dimensional. The results of both epidemiologic and laboratory research indicate that even if single pollutants can dominate certain effects when multiple air pollutions co-exist, their overall toxicity may differ from that found in investigations specific to individual pollutants [16]. The World Health Organization also focused on “Multi-pollutant effect estimates as a basis for joint health impact assessment” in the discussions for updating the global air quality guidelines [17]. Now is the time to shift the emphasis of air pollution health research toward a more comprehensive, forward-looking, multipollutant perspective in view of the increasing trend toward multipollutant regulatory strategies.

In fact, to make up for the lack of studying health risks from the perspective of single pollutants, some scholars have studied the health effects of multiple pollutants. The general idea of these methods is to estimate each single pollutant effect while controlling for the presence of the others, and then define the multi-pollutant effect as the sum of the effects of each air pollutant [18,19,20], regardless of interactions or nonlinear effects. Since interactions among ambient air pollutants are plausible, partially false conclusions would have been reached by estimating the effects of each pollutant separately and adding them up [16]. In addition, even if there is research that can define similar statistical models to account for higher-order interaction, so as to capture the health burden associated with simultaneous exposure to more than two pollutants [10,21], when some highly correlated pollutants are simultaneously included in the regression model, the results can become highly unstable and often inaccurate [22]. Therefore, disentangling the health effect of multi-pollutants has been a long-discussed challenge.

Considering these problems, instead of directly introducing pollutant concentrations, we classified complex air pollution into different types based on different predominant pollutants and transformed them into a set of predictors to estimate mortality risk for different pollution types. At the same time, due to China’s vast territory and regional differences in climate and emission structure, it is necessary to conduct studies in multiple cities with significant climate differences. Eight large Chinese cities with a population of more than 3 million and located in different climate regions were selected to investigate the relationship between complex air pollution and mortality, with a view to strengthening the capacity of coordinated, health-led air pollution control based on local conditions and providing a basis for the formulation of multi-pollution air quality standards that meet local needs.

## 2. Methods

### 2.1. Study Area

A multicity time-series study was conducted to assess the adverse effects of short-term exposure to high single-pollutant or high multi-pollutant types on daily mortality in eight metropolises in China. The cities, which were not chosen randomly but because there were relatively complete data in each and these are large cities with a population of more than 3 million [23] in different climate regions of China, were Changchun, in the Jilin province (short for CC, temperate continental monsoon climate, Northeast China), Urumqi, in Xinjiang Uygur Autonomous Region (short for WLMQ, temperate continental arid and semi-arid climate, west of Northwest China), Beijing, the capital of China (short for BJ, warm temperate semi-humid climate, North China), Xian, in the Shaanxi province (short for XA, temperate semi-arid monsoon climate, east of Northwest China), Nanjing, in the Jiangsu province (short for NJ, the transition zone between warm temperate zone and subtropical zone, East China), Wuhan, in the Hubei province (short for WH, subtropical humid monsoon climate, Central China), Kunming, in the Yunnan province (short for KM, subtropical, tropical plateau monsoon climate, Southwest China), and Guangzhou in the Guangdong province (short for GZ, subtropical—tropical humid monsoon climate, South China).

### 2.2. Data Collection

For the period 2013–2016, daily mortality counts for all nonaccidental causes (International Classification of Diseases, Revision 10 (ICD-10, A00-R99)) in each district of the eight cities were obtained from the Chinese Center for Disease Control and Prevention. Based on the district code, the total number of non-accidental deaths per day for each city was calculated.

Air pollutant concentration observations including ozone, nitrogen dioxide, and particulate matter with an aerodynamic diameter of <2.5 µm (PM_2.5_) were acquired from the Ministry of Ecology and Environment of the People’s Republic of China. After hourly values greater than 500 μg/m^3^ were converted into lacking values to protect against outliers [24], the daily mean values of PM_2.5_ and NO_2_ and the 1-h maximum ozone at each monitor were calculated. For assessing the population exposure level in a city, a single monitoring station is unlikely to be sufficient [25]. To reduce random errors, multiple-site averages for a city were applied to reflect the population’s exposure risk [26,27]. These daily, within-city average concentrations were used as the average exposure of the population at risk in each city.

Daily average meteorologic data regarding mean temperature, relative humidity, air pressure, wind speed, and precipitation were obtained from the China Integrated Meteorological Information Service System of National Meteorological Information Center, China Meteorological Administration. Because the variability of mean temperature and relative humidity within the city limits is small [28], the weather conditions for each city were derived entirely from one monitoring station there.

### 2.3. Category of Pollution Types

To estimate the health effects of multiple pollutants, we selected the pollutants in this study. According to WHO [17], PM, O_3_, NO_2_, and SO_2_ are pollutants in its “Group 1”, which “should be considered of greatest importance in the process of updating the WHO Air Quality Guidelines”. Since PM_2.5_ and PM_10_ are strongly correlated, PM_2.5_, which with smaller particle size and is more harmful to human health [29], is chosen to represent PM in the current study. Considering that the emissions of SO_2_ have been drastically cut in recent years [30], in this paper, PM_2.5_, O_3_, and NO_2_ were selected to evaluate the health effects of complex air pollution.

Similar classification and grouping methods have been used in previous environmental health studies. Dimensionality reduction by grouping is an effective idea in the latest statistical research on the impact of multiple pollution-related exposures on human health. It transforms a large number of correlated variables (pollutants, exposures, etc.) into a set of independent factors to estimate health outcomes [31]. Meanwhile, previous studies on the health effects of pollutants or other factors often used the method of stratified study, set cutoff points for the study variables, and studying the difference in health effects at different levels above and below the cutoff point to study the heterogeneity [32,33,34]. So, we created a variable with eight levels comprising every combination of levels above and below the cutoff point of the three pollutants mentioned above, that is high and low levels of PM_2.5_, NO_2_, and O_3_, and used this variable as the primary exposure in the model. The specific definition is as follows.

First of all, in order to give consideration to sufficient data and appropriate discrimination between high and low pollution levels, we took the median of PM_2.5_ and NO_2_ in each city and the 70th quartile of O_3_ (Table 1) as the cut-off point and divided the three pollutants into low and high levels respectively. The reason why the 70th quartile of O_3_ is used instead of the median as the cut-off point is that the distribution of ozone concentration is asymmetric, which is more distributed in the range of low concentration. Using the median as a cut-off will make a large number of observations concentrated at the cut-off point, which is difficult to distinguish between high and low concentration, resulting in bias. The cut-off is different for each city, and its values are shown in Table 1.

Then a set of permutations of these three pollutants at different levels were grouped as eight different types (Table 2), including one reference type, three single-pollutant types and four multi-pollutant types. They are Type 0 (reference type): the concentrations of the three pollutants are all below the cut-off; Type 1 (high single-pollutant type): PM_2.5_ concentration is above the cut-off, while the concentration of the other two pollutants is below the cut-off; Type 2 (high single-pollutant type): NO_2_ concentration is above the cut-off, while the concentration of the other two pollutants is below the cut-off; Type 3 (high single-pollutant type): O_3_ concentration is above the cut-off, while the concentration of the other two pollutants is below the cut-off; Type 4 (high multi-pollutant type): PM_2.5_ and NO_2_ concentration is above the cut-off, while the concentration of O_3_ is below the cut-off; Type 5 (high multi-pollutant type): PM_2.5_ and O_3_ concentration is above the cut-off, while the concentration of NO_2_ is below the cut-off; Type 6 (high multi-pollutant type): O_3_ and NO_2_ concentration is above the cut-off, while the concentration of PM_2.5_ is below the cut-off; Type 7 (high multi-pollutant type): the concentrations of the three pollutants are all above the cut-off.

**Figure 1:**
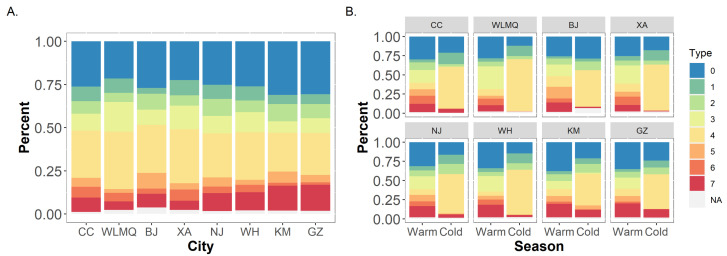
Frequency distribution of different pollution types in 8 cities ((**A**) all-year results; (**B**) seasonal results). The type of pollution and the pollutant classified as high level in this type are as follows, 0: reference type, none; 1: only PM_2.5_; 2: only NO_2_; 3: only O_3_; 4: PM_2.5_ and NO_2_; 5: PM_2.5_ and O_3_; 6: NO_2_ and O_3_; 7: PM_2.5_, NO_2_ and O_3_; NA: lacking values.

Types containing conditions with ozone concentrations above the cut-off occurred very infrequently (less than 20 times) during the cold season in some cities (e.g., Type 3, 5, 6, and 7 in BJ) (Figure 1). Since too few data would lead to instability in the model, these types with too few occurrences were excluded from the seasonal analyses of these cities.

### 2.4. Data Analysis

Because daily mortality counts typically follow a Poisson distribution, we used a generalized additive model (GAM) with a Poisson link to evaluate the association between mortality and air pollution types controlling for average temperature, relative humanity, seasonality, and long-term trends using cubic smoothing spline [35,36]. We created a variable with 8 levels representing every combination of three pollutant concentration categories (high or low levels of PM_2.5_, NO_2_, and O_3_) in Section 2.3, and used this variable as the main exposure in the model:

(1) $$\log\left[E\left(Y_{k}\right)\right]=\alpha +DOW+\beta_{k}\times X_{k}+s\left(time,df\right)+s\left(temperature,\;df\right)+s\left(RH,df\right)$$

where $E\left(Y_{k}\right)$ is the expected number of deaths on day t. α is the model intercept. $X_{k}$ is the categorical variable created in Section 2.3, representing the pollution types with different levels of three pollutants. Type 0 (PM_2.5_, NO_2_, and O_3_ are all at low levels) was used as a reference type to calculate parameter estimates for the seven different high-level pollution types. $\beta_{k}$ is the regression coefficient for $X_{k}$. Day of the week was also included as a dummy variable: DOW. time represents time to adjust for long-term trends and seasonality. s(time, df), s(temperature, df), and s(RH, df) were spline smoothers for date, daily average temperature, and daily average relative humidity, respectively, which captures the nonlinear relationships of the covariates of daily mortality with time trend and the weather parameters. df is the degree of freedom determined by minimizing the Akaike’s Information Criterion (AIC). Considering that similar studies have all used a degree of freedom below 10. In this study, tests are conducted in the range of 4 or 8 (1–2 per year) for each time term, and the degrees of freedom of the model with the lowest AIC is selected. The degrees of freedom of average temperature and average relative humidity was 4.

Lag structures are included as air pollution may affect health outcomes happening on the same day or on subsequent days. We analyzed the one-day lag mode from Lag0 to Lag5, where Lag0 represented the pollution type on day 0, Lag1 was the pollution type on the previous day, and so on. In addition to the overall analyses, all models were also stratified by season (cooler vs. warmer months). The cold season was defined as November through March and the warm season as April through October.

All results were presented as relative risks (RRs) or excess risk (ER) of mortality and their 95% Confidence Interval (95%CI), calculated from the relative risk (RR) and excess risk (ER) as follows:

(2) $$\mathrm{RR}=e^{\beta }$$

ER = (RR − 1) × 100(3)

All statistical tests were 2-sided, and *p*-values < 0.05 were considered statistically significant. The analysis was performed in R-software, version 4.1.0, using time-series analysis with the mgcv package.

### 2.5. Sensitivity Analyses

**Figure A1:**
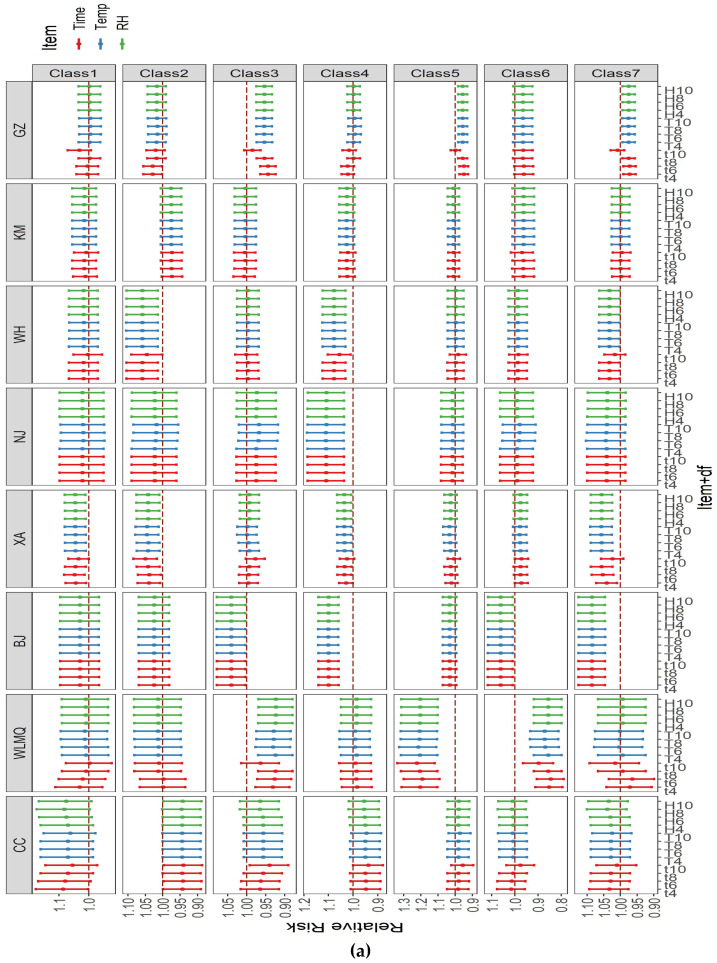

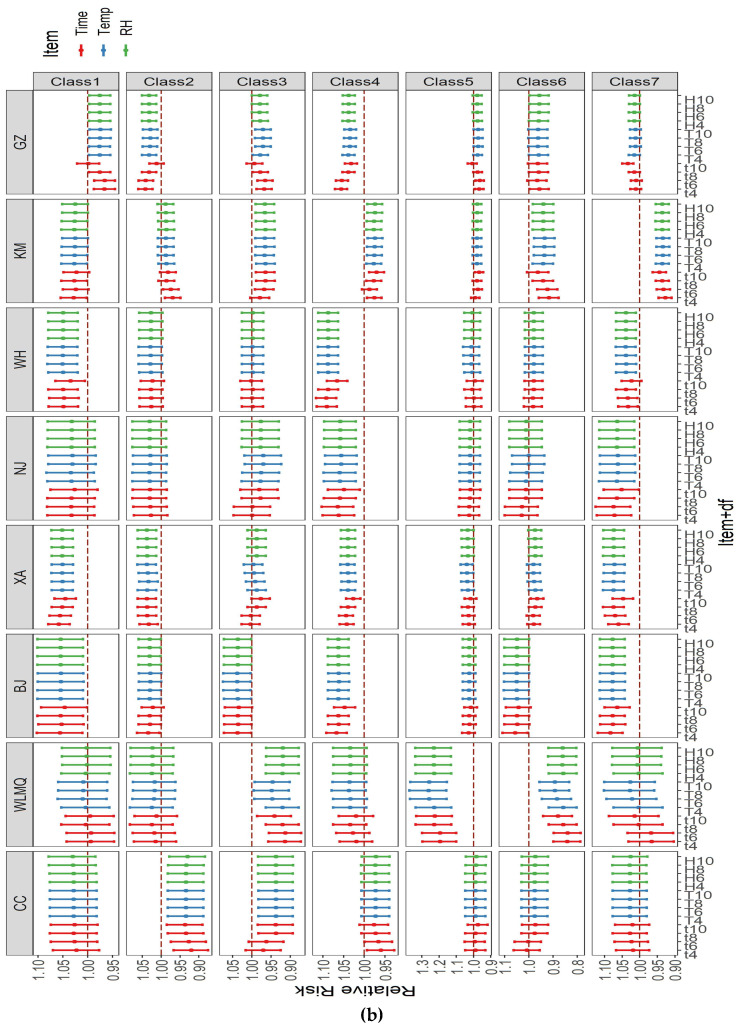

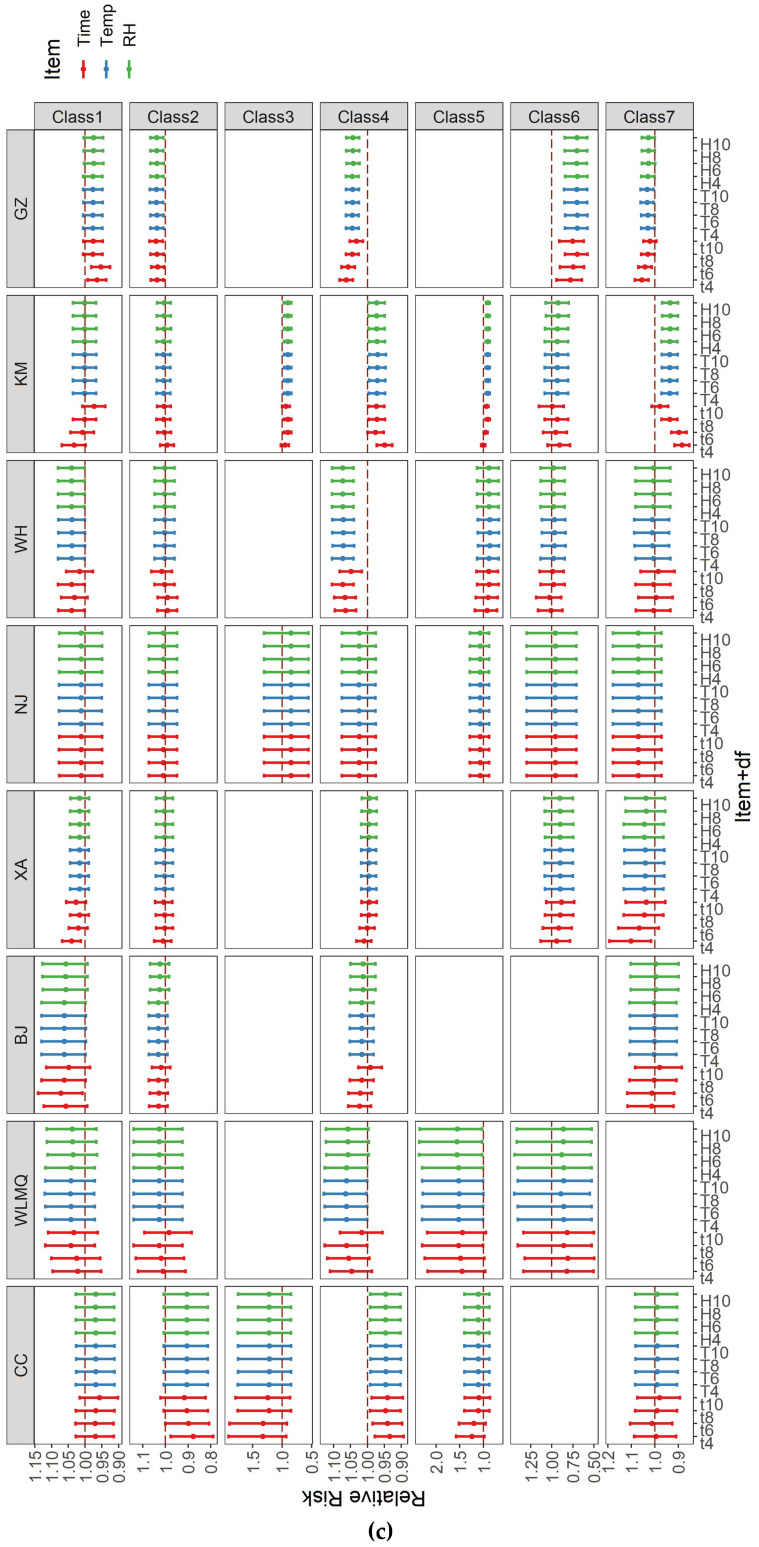
A change in the number of degrees of freedom at intervals of 2 within a range of 4 to 10 df, for time trend, temperature, and relative humidity respectively, during all-year (**a**), warm season (**b**), and cold season (**c**).

Finally, sensitivity analysis was performed to ensure the stability of the model. Within a range of 4 to 10 df, a change in the number of degrees of freedom at intervals of 2 for time trend did not substantially affect the estimated effects of each pollutant type (Figure A1). We also compared the effects of each pollutant type with alternative values for degrees of freedom for meteorological conditions. Within a range of 4 to 10 df, a change in the number of degrees of freedom at intervals of 2 for temperature and relative humidity resulted in almost identical estimated effects of air pollution on all-cause mortality (Figure A1). In this respect, our findings were relatively robust.

## 3. Results

Table A1 shows the summary statistics of daily all-cause mortality, air pollution, and meteorological variables for each pollution type during the study period. Overall, among all the types, there was average mortality from 49.5 ± 34.0 (type 6) to 65.0 ± 40.2 (type 7) person/day in all eight cities from 2013 to 2016. During the period, the highest daily average concentration of PM_2.5_ and NO_2_ were type 4 (113.8 ± 71.3 and 67.4 ± 20.9 µg/m^3^ respectively). The highest daily 1-h maximum O_3_ was type 5 (175.1 ± 47.5 µg/m^3^). In total, the highest average daily temperature and relative humidity were type 3 (24.8 ± 4.6 °C) and type 1 (71.7 ± 17.2%), and the lowest were type 4 (5.9 ± 10.3 °C) and type 6 (57.3 ± 17.4%), respectively.

We made statistics on the frequency and ratio of high single-pollutant type and high multi-pollutant type, as well as the frequency of each pollution type in each city. In general, multi-pollutant types (type 4, type 5, type 6, and type 7) occur more frequently than single-pollutant types (type 1, type 2, and type 3). The frequency ratio of multi-pollutant types to single-pollutant types was the largest in Beijing, the frequency of multi-pollutant types was 2.22 times that of single-pollutant types there. While the ratio was the smallest in Urumqi, it is 1.48 times of frequency of single-pollutant types (Table 3). The frequency of each pollution type was different during the study period (Figure 1). In the whole year, the results in all eight cities showed that type 4, the high multi-pollutant type with a higher concentration level of PM_2.5_ and NO_2_, was the most frequent pollution type. During the study period, the frequency of type 4 in eight cities ranged from 33.3% (486 days, Urumqi) to 24.4% (327 days, Kunming). The next type with high frequency was mainly type 7 (PM_2.5_, NO_2_ and O_3_ are at high levels) in southern cities, ranging from 15.1% (220 days, Guangzhou) to 10.3% (151 days, Nanjing), while type 3 (only O_3_ is at high level) in northern cities, ranged from 17.2% (251 days, Urumqi) to 8.9% (130 days, Beijing). In the warm season, the most frequent pollution types are type 3 and type 7, both of which include ozone as a predominant pollutant. In the cold season, the frequency of type 4 (PM_2.5_ and NO_2_ are at high levels) almost accounted for half of the whole cold season, which was the type with the highest frequency in all eight cities.

**Figure 2:**
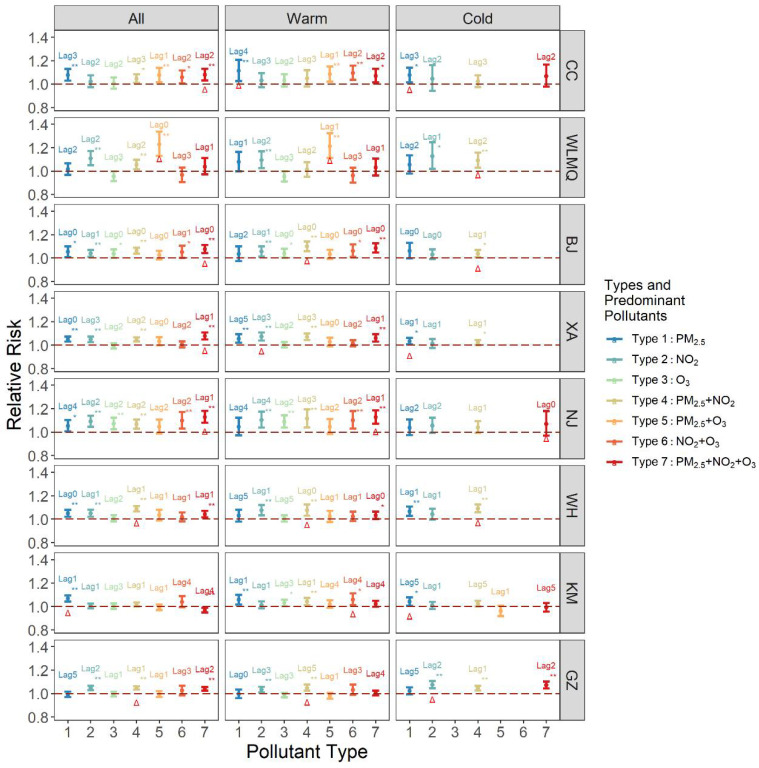
Association between different pollution types and RRs (95% CIs) of mortality modified by season. Among them, types 1–3 are high single-pollutant types, and types 4–7 are high multi-pollutant types. Considering the lag of lag0 to lag5, the day with the highest RR value of each type was selected for analysis and comparison, and the lag day was marked above the corresponding type. The red triangle is indicated as the type with the highest RR, and the results that pass the significance test are preferentially selected. The eight cities are arranged north to south by latitude from top (CC) to bottom (GZ). * *p* < 0.05, ** *p* < 0.01.

By comparing the greatest RR along lag0–lag5 of each pollution type (Figure 2), we identified the pollution types with the highest mortality risk throughout the year and in different seasons in each city (Table 4, Table 5 and Table 6). In all-year analyses, we found that the pollution types with the highest RRs in 7 cities except Kunming all belong to high multi-pollutant pollution types, that are type 7 (high O_3_, PM_2.5_, and NO_2_) and type 4 (high PM_2.5_ and NO_2_). In all eight cities, half of the types with the highest risks in each city were type 7, and the highest RR was 1.129 (1.080, 1.181) in Nanjing, and the mortality effect of type 7 was significant in all six cities except Kunming and Urumqi. In addition, type 4 was also significantly associated with death in 7 cities, with the maximum RR of 1.089 (1.066, 1.113) in Wuhan. Results from lag models indicated that exposure to high multi-pollutant air pollution on more recent days, such as from the same day to 2 days ago was associated with a larger risk of mortality than exposure on less recent days (such as three days ago or earlier). In terms of seasons, high multi-pollutant pollution types have a higher risk in most cities in the warm season than in the cold season. During the warm season, types 4 and 7 were most significantly associated with death, and type 6 (high O_3_, and NO_2_) was significantly associated with death in half of the cities. During the cold season, most of pollution types had the highest RR values of type 1 and type 4, and type 4 passed the significance test more than type 1.

**Figure 3:**
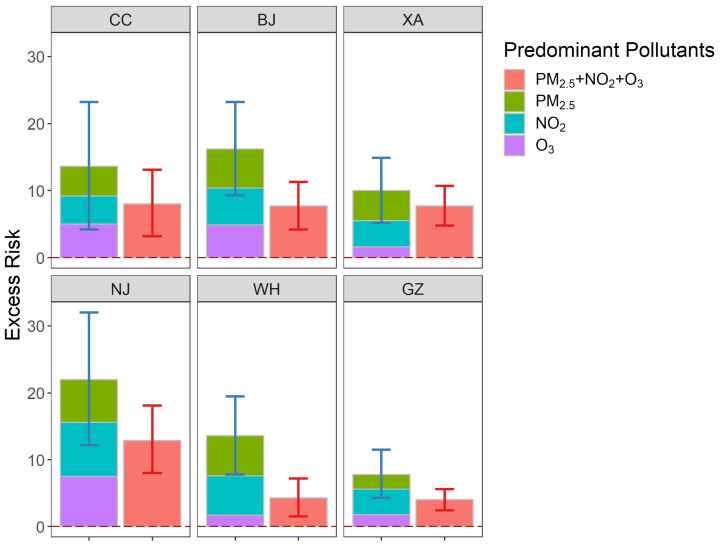
Comparison of the simple sum of the excess risks (95% CIs) of individual pollutants (PM_2.5_, NO_2_, and O_3_) at high levels (left) with the excess risks (95% CIs) of the multi-pollutant air pollution type with all three pollutants are simultaneously at high levels (right).

Furthermore, we compared the simple sum of the excess risks of individual pollutants at high levels with the excess risks of the multiple pollution type with all three pollutants simultaneously at high levels. The concentration levels of other pollutants are not taken into account when calculating the exposure risk at high levels for each pollutant alone. While calculating the impact of combined exposure, the concentration level of three pollutants is all considered, and the risk is calculated when the three pollutants are at high levels simultaneously. The results for each type of excess risk higher than 0 were listed in Figure 3. The results showed that combined effects that were less than simple additive.

## 4. Discussion

In this study, we evaluated the adverse effects of complex air pollution. A variable containing eight levels of different combinations of concentrations at high or low levels of PM_2.5_, O_3_, and NO_2_ was created to characterize the air pollution characteristics of different types of air pollution. Using this variable as the main exposure in GAM, we investigated the association between mortality risk and atmospheric composite pollution in eight large cities with different climate zones in China. In our analysis, we found evidence that exposure to high multi-pollutant types which several pollutants with high concentrations simultaneously was linked to a higher relative risk than exposure to high single-pollutant types. In the whole year, the high multi-pollutant type with high PM_2.5_, NO_2_, and O_3_ and the high multi-pollutant type with high PM_2.5_ and NO_2_ were more associated with death, and the highest RRs were 1.129 (1.080, 1.181) and 1.089 (1.066, 1.113), respectively. In addition, the pollution types that most threaten people are different in different cities. In terms of seasons, the risk of complex air pollution is greater in most cities in the warm season than in the cold season. In addition, the results also showed that the excess risk from simultaneous exposure to multiple pollutants was less than the sum of individual air pollutants effects.

The results of the present study indicate that type 7 (high PM_2.5_, O_3_, and NO_2_) and type 4 (high PM_2.5_ and NO_2_), the two high multi-pollutant types, had the highest relative risks. Meanwhile, the association between different types of complex air pollution and death varied between regions. This means that the high multi-pollutant pollution type is more associated with death than the high single-pollutant pollution type, the problem of complex air pollution to health remains grim. The health burden of multiple pollutants has also been studied in other countries and in individual cities in China. The health effects of multi-pollutant air pollution in different cities may different due to the differences in emission sources and pollutant components in different cities. Papathomas et al. [37] assessed the combined effect of environmental factors on carcinogenesis in Europe and found that higher exposure to both NO_2_ and PM_10_ and residential proximity to roads were more common in high-risk populations. In a study in the United States, Wesson et al. [38] compared the single-pollutant control strategy with the “Multi-pollutant, Risk-based” control strategy, and found that the latter greatly reduced the per-person emissions of PM_2.5_ and O_3_, and had greater health benefits. In China, Huang et al. [39] selected PM_2.5_, NO_2_, O_3_ and SO_2_ as the air pollutant mixture to examine the daily contribution of air pollutants to the risk of outpatient visits in Guangzhou and found that NO_2_ and O_3_ made prominent contribution. Zhu et al. [40] noted that the primary type of high multi-pollutant air pollution in Tianjin in 2020 was PM_2.5_-NO_2_ co-pollution. This is similar to our results, suggesting that policymakers should shift to a multi-pollutant approach to air quality and achieve greater public health protection through the regulation of multiple sources of air pollution and the overall mixture air pollution.

Overall, in terms of predominant pollutants, the types with the highest RR in all cities included high levels of PM_2.5_. Traini et al. [41] in Dutch observed positive associations between air pollution mixtures and mortality, PM_2.5_ is the main driver of the associations. According to the most polluted country and region ranking based on annual average PM2.5 concentration in 2021, China belongs to one of the World’s most polluted countries [42], and Yan et al. [43] found that evidence of the association between PM_2.5_ and the risk of cardiovascular death was higher during periods with high PM_2.5_ concentration than during periods with low PM_2.5_ concentration. In addition, vehicle emissions are a major source of NO_2_, which is an important precursor to PM_2.5_ and has complex links to it. At the same time, due to the robust positive correlation between PM_2.5_ and NO_2_, type 4 (high PM_2.5_ and NO_2_) is the most frequent multi-pollutant type and has the most significant association with mortality.

Additionally, different cities have different outdoor activities patterns and ventilation habits in different seasons due to each climate feature, which will affect indoor and outdoor exposure rates and thus affect health. The results showed that type 7, which PM_2.5_, NO_2_ and O_3_ were all at high levels, has the highest risk in Nanjing and the cities to the north of it, while in Wuhan and Guangzhou to the south of Nanjing, the highest risk was type 4, that is, PM_2.5_ NO_2_ were at high levels and O_3_ was at low level. This may be related to the different exposure types of urban residents with different climate features. The Severe cold in the cold season in the north and heatwave and heavy rain in the warm season in the south will reduce local people’s exposure to pollutants outdoors. Moreover, the seasonal variation of ozone is obvious, and its concentration is much higher in the warm season than in the cold season. In the Pearl River Delta region, however, the cold season is cool and dry, with little temperature change, and people are more likely to go outside and open their windows for ventilation, thus exposing themselves to higher levels of air pollution. While the warm season is hot and humid, thus people often use air conditioning, which reduces the risk of exposure to ambient air pollution [44]. This lifestyle will reduce the outdoor ozone exposure of people in southern China.

The results also revealed that the risk of complex air pollution is greater in most cities in the warm season than in the cold season. This result may be caused by the interaction between meteorological conditions and pollutants in addition to the differences in population activity patterns in different seasons. Studies in Germany, Portugal, and Italy have shown that the increased risk of death due to elevated pollutant concentrations is more dramatic at high temperatures than at low temperatures [45,46]. At the same time, research results in China also show that extreme high temperature will increase the risk of death of pollutants such as PM2.5 and PM10, while the effect of extreme low temperature is lower than that of extreme high temperature [47,48,49,50]. Therefore, the modifying effects of high temperature on pollutants may be the reason why the mortality risk of high multi-pollutant types is higher in the warm season.

Rather than following the type of previous air pollution health studies that looked at individual pollutants and add up the effects of them together, we consider air pollution as a mixture to identify the mortality risk of the complex pollution of different dominant pollutants in China. This approach avoids the problem of overestimating the combined effect due to possible collinearity and interaction when the effect of individual air pollutants is summed. Furthermore, the research covers a wide range of 8 major cities in China, which are located in different regions with different characteristics of climate, pollution level and economic development level. These cities have strong regional representation which makes the results of this study more comprehensive than those of a single city. In summary, this study provides a reference for putting forward multi-pollutant control strategies for air quality following local conditions, to strengthen the ability of health-driven coordinated air pollution control in China.

There are still some limitations to the present study. Firstly, 8 cities with large climate differences were selected nationwide for research, which has regional representativeness to a certain extent, but its representativeness is still limited, and there may be some deviations in direct application to other cities. Secondly, as a time series analysis, this study inevitably has exposure errors. Since it is difficult to obtain the true exposure of individuals, observations from monitoring stations are used as proxies for population exposure, which leads to a certain degree of exposure error. Finally, due to data limitations, we did not classify the population by gender, age, economy, and education level, so we could not put forward more targeted health suggestions for vulnerable populations.

## 5. Conclusions

This paper confirms the robust health hazards of complex air pollution and suggests that the mortality risk from exposure to the high multi-pollutant type is generally higher than that of the high single-pollutant type and varies regionally and seasonally. Type 7 with high level of all three pollutants (PM_2.5_, O_3_, and NO_2_) and type 4 with high level of PM_2.5_ and NO_2_ have a greater relative risk than other pollution types. In addition, the pollution types that most threaten people are different in different cities. In terms of seasons, the risk of complex air pollution is greater in most cities in the warm season than in the cold season. The results also showed that the excess risk from simultaneous exposure to multiple pollutants was less than the simple sum of individual air pollutants effects. Calculating the effect of individual air pollutants separately and adding them together may lead to an overestimation of the combined effect. Therefore, the focus of air pollution health research needs to shift to a multi-pollutant perspective that considers air pollution as a whole rather than separately.

## Figures and Tables

**Table 1:** The cut-off of PM_2.5_, NO_2_, and O_3_ in each city (μg/m^3^).

City	Median of PM_2.5_	Median of NO_2_	70th-Quartile of O_3_ (1h-Max)
Changchun	43.667	39.925	113.000
Urumqi	49.146	50.967	89.514
Beijing	62.208	46.903	138.450
Xian	58.075	44.782	119.400
Nanjing	54.501	45.483	137.450
Wuhan	61.625	45.789	142.660
Kunming	27.954	29.043	105.667
Guangzhou	38.051	44.233	140.600

**Table 2:** The 8 types of pollution and the predominant pollutants (the pollutant whose concentration is higher than the cut-off).

Pollution Type	Predominant Pollutants
0	None
1	PM_2.5_ only
2	NO_2_ only
3	O_3_ only
4	PM_2.5_ + NO_2_
5	PM_2.5_ + O_3_
6	NO_2_ + O_3_
7	PM_2.5_ + NO_2_ + O_3_

**Table 3:** Percentage and ratio of the frequency of multi-pollutant and single-pollutant types.

City	Single-Pollutant Type (%)	Multi-Pollutant Type (%)	The Ratio of the Frequency (Multi-Pollutant/Single-Pollutant)
Changchun	25.5	47.2	1.851
Urumqi	30.7	45.4	1.479
Beijing	21.5	47.8	2.223
Xian	28.7	46.6	1.624
Nanjing	28.2	44.9	1.592
Wuhan	26.6	45.2	1.699
Kunming	22	45	2.045
Guangzhou	22.5	45.1	2.004

**Table 4:** The pollution type with the highest lag of 0–5 days and their corresponding risk values and lag days in 8 cities. Pollutants at high levels are listed in parentheses (Annual). ** *p* < 0.01.

City	The Pollution Type with the Highest RR (Annual)	The Highest RR (with 95% CIs)	Lag
Changchun	Type 7 (O_3_ + PM_2.5_ + NO_2_)	1.080 (1.032, 1.131)	lag2 **
Urumqi	Type 5 (O_3_ + PM_2.5_)	1.228 (1.129, 1.336)	lag0 **
Beijing	Type 7 (O_3_ + PM_2.5_ + NO_2_)	1.077 (1.042, 1.113)	lag0 **
Xian	Type 7 (O_3_ + PM_2.5_ + NO_2_)	1.077 (1.048, 1.107)	lag0 **
Nanjing	Type 7 (O_3_ + PM_2.5_ + NO_2_)	1.129 (1.080, 1.181)	lag1 **
Wuhan	Type 4 (PM_2.5_ + NO_2_)	1.089 (1.066, 1.113)	lag1 **
Kunming	Type 1 (PM_2.5_)	1.069 (1.042, 1.096)	lag1 **
Guangzhou	Type 4 (PM_2.5_ + NO_2_)	1.049 (1.034, 1.063)	lag1 **

**Table 5:** The pollution type with the highest lag of 0–5 days and their corresponding risk values and lag days in 8 cities. Pollutants at high levels are listed in parentheses (Warm Season). ** *p* < 0.01.

City	The Pollution Type with the Highest RR (Warm Season)	The Highest RR (with 95% CIs)	Lag
Changchun	Type 1 (PM_2.5_)	1.113 (1.028, 1.206)	lag4 **
Urumqi	Type 5 (O_3_ + PM_2.5_)	1.214 (1.112, 1.324)	lag1 **
Beijing	Type 4 (PM_2.5_ + NO_2_)	1.099 (1.059, 1.141)	lag0 **
Xian	Type 2 (NO_2_)	1.073 (1.040, 1.108)	lag3 **
Nanjing	Type 7 (O_3_ + PM_2.5_ + NO_2_)	1.127 (1.071, 1.186)	lag1 **
Wuhan	Type 4 (PM_2.5_ + NO_2_)	1.077 (1.031, 1.124)	lag0 **
Kunming	Type 6 (O_3_ + NO_2_)	1.060 (1.009, 1.112)	lag4 **
Guangzhou	Type 4 (PM_2.5_ + NO_2_)	1.049 (1.023, 1.076)	lag5 **

**Table 6:** The pollution type with the highest lag of 0–5 days and their corresponding risk values and lag days in 8 cities. Pollutants at high levels are listed in parentheses (Cold Season). * *p* < 0.05, ** *p* < 0.01.

City	The Pollution Type with the Highest RR (Cold Season)	The Highest RR (with 95% CIs)	Lag
Changchun	Type 1 (PM_2.5_)	1.077 (1.015, 1.142)	lag3 *
Urumqi	Type 4 (PM_2.5_ + NO_2_)	1.091 (1.028, 1.157)	lag2 **
Beijing	Type 4 (PM_2.5_ + NO_2_)	1.037 (1.008, 1.068)	Lag4 *
Xian	Type 1 (PM_2.5_)	1.032 (1.005, 1.061)	Lag1*
Nanjing	Type 7 (O_3_ + PM_2.5_ + NO_2_)	1.070 (0.971, 1.178)	lag0
Wuhan	Type 4 (PM_2.5_ + NO_2_)	1.092 (1.059, 1.125)	lag1 **
Kunming	Type 1 (PM_2.5_)	1.041 (1.005, 1.078)	lag5 *
Guangzhou	Type 2 (NO_2_)	1.076 (1.045, 1.107)	lag2 **

## Data Availability

All data generated or analyzed during this study are included in this article.

## Appendix A

**Table A1:** Annual average of daily mean concentrations (standard deviation) for air pollution, meteorological variables, and daily all-cause mortality for each pollution type in 8 Chinese cities, 2013–2016. The bold represent the statistics of the pollution types with the highest all-cause deaths and pollutant concentrations in each city.

City	Type	All (Mean ± SD)	O_3__1h (Mean ± SD, µg/m^3^)	PM25 (Mean ± std, µg/m^3^)	NO_2_ (Mean ± std, µg/m^3^)	TEM (Mean ± std, °C)	RH (Mean ± std, %)
All	0	62.5 ± 40.9	76.6 ± 28.4	27.2 ± 12.0	30.3 ± 8.3	15.5 ± 10.1	69.4 ± 21.0
	1	60.5 ± 42.7	71.3 ± 31.6	73.3 ± 33.2	36.0 ± 7.8	9.0 ± 11.1	71.7 ± 17.2
	2	63.3 ± 42.5	74.0 ± 29.5	37.3 ± 12.2	50.8 ± 11.2	13.5 ± 8.7	66.6 ± 19.4
	3	53.8 ± 36.6	157.9 ± 41.1	32.8 ± 11.2	33.2 ± 7.9	24.8 ± 4.6	61.6 ± 16.5
	4	64.2 ± 44.5	63.7 ± 32.5	**113.8 ± 71.3**	**67.4 ± 20.9**	5.9 ± 10.3	69.6 ± 15.2
	5	56.7 ± 35.2	**175.1 ± 47.5**	70.9 ± 31.3	34.0 ± 8.2	23.8 ± 4.9	64.6 ± 15.5
	6	49.5 ± 34.0	163.1 ± 40.5	39.1 ± 11.0	53.0 ± 9.8	22.7 ± 4.3	57.3 ± 17.4
	7	**65.0 ± 40.2**	170.9 ± 46.5	80.8 ± 41.5	60.3 ± 18.2	20.3 ± 5.8	63.2 ± 16.9
BJ	0	49.0 ± 10.0	83.8 ± 25.5	25.9 ± 14.8	30.9 ± 9.7	11.8 ± 10.5	41.7 ± 22.3
	1	49.9 ± 10.5	76.3 ± 28.3	92.6 ± 38.5	40.5 ± 5.0	11.7 ± 10.5	62.1 ± 21.5
	2	50.9 ± 9.0	72.8 ± 29.6	44.6 ± 11.5	57.2 ± 7.5	9.6 ± 9.4	45.0 ± 15.4
	3	44.8 ± 7.5	195.5 ± 40.5	38.2 ± 13.9	35.2 ± 6.8	25.7 ± 3.1	56.0 ± 13.7
	4	**52.8 ± 8.8**	54.4 ± 36.5	**146.9 ± 74.6**	**81.1 ± 22.5**	6.6 ± 8.2	61.6 ± 17.2
	5	44.1 ± 7.8	**216.8 ± 43.2**	101.3 ± 30.4	36.6 ± 5.9	26.3 ± 3.4	66.4 ± 11.7
	6	45.9 ± 6.2	196.0 ± 38.7	45.8 ± 11.7	55.8 ± 6.6	24.8 ± 3.6	45.1 ± 12.9
	7	46.8 ± 7.3	203.3 ± 47.5	121.1 ± 52.4	64.7 ± 17.0	21.8 ± 4.4	56.2 ± 12.5
CC	0	20.8 ± 6.5	79.4 ± 18.4	26.1 ± 9.4	29.2 ± 6.1	7.1 ± 12.4	63.9 ± 17.1
	1	**21.9 ± 6.6**	76.5 ± 19.4	63.2 ± 22.7	33.2 ± 5.9	−2.9 ± 11.8	60.0 ± 15.1
	2	18.9 ± 5.8	86.4 ± 18.8	31.8 ± 8.5	46.4 ± 6.3	11.6 ± 9.8	59.5 ± 16.1
	3	19.7 ± 6.8	142.5 ± 26.7	27.5 ± 8.4	31.1 ± 5.5	21.6 ± 4.5	62.2 ± 15.9
	4	20.6 ± 6.7	69.9 ± 19.7	**107.8 ± 65.2**	56.2 ± 13.0	−5.6 ± 9.9	63.3 ± 13.9
	5	19.9 ± 7.0	**155.2 ± 21.8**	66.6 ± 20.6	30.6 ± 6.2	21.3 ± 5.6	57.4 ± 19.4
	6	21.0 ± 6.5	146.9 ± 26.2	31.2 ± 7.4	47.9 ± 7.1	21.1 ± 4.0	57.0 ± 15.2
	7	21.3 ± 6.6	148.1 ± 30.7	96.9 ± 60.9	**58.4 ± 14.9**	14.0 ± 8.9	51.9 ± 15.6
GZ	0	124.7 ± 23.6	77.7 ± 36.2	23.1 ± 7.1	32.4 ± 6.7	22.5 ± 6.5	81.7 ± 10.1
	1	121.4 ± 21.5	90.3 ± 36.3	50.3 ± 11.4	35.4 ± 6.6	18.6 ± 6.7	73.9 ± 12.8
	2	130.7 ± 23.1	61.4 ± 35.3	30.0 ± 4.8	51.7 ± 6.3	21.1 ± 5.1	86.2 ± 8.4
	3	115.6 ± 17.3	178.6 ± 36.3	27.1 ± 5.2	32.6 ± 5.5	28.6 ± 1.4	78.3 ± 5.2
	4	**134.4 ± 26.3**	79.6 ± 35.2	**66.3 ± 23.5**	**68.5 ± 19.3**	17.7 ± 4.8	78.7 ± 11.8
	5	114.4 ± 14.0	**199.1 ± 43.3**	50.0 ± 11.1	39.5 ± 3.9	27.3 ± 2.4	75.5 ± 8.3
	6	114.7 ± 17.7	186.8 ± 38.9	32.7 ± 5.3	50.2 ± 7.0	25.3 ± 2.8	81.8 ± 10.8
	7	122.5 ± 20.1	196.9 ± 46.7	63.8 ± 20.7	64.3 ± 19.6	23.4 ± 4.7	77.7 ± 7.9
KM	0	85.8 ± 17.3	71.6 ± 19.5	18.1 ± 4.7	21.9 ± 4.5	17.6 ± 4.4	74.0 ± 13.5
	1	98.1 ± 22.8	79.9 ± 18.0	35.1 ± 6.5	23.9 ± 4.4	13.6 ± 5.5	71.9 ± 13.5
	2	87.6 ± 21.6	71.5 ± 20.3	22.6 ± 4.0	33.6 ± 3.7	15.2 ± 4.2	74.5 ± 13.4
	3	85.2 ± 13.9	120.3 ± 16.4	22.5 ± 3.6	21.5 ± 4.6	19.8 ± 3.2	64.2 ± 13.6
	4	**96.7 ± 24.0**	74.1 ± 21.5	43.8 ± 13.0	40.1 ± 7.6	11.8 ± 4.4	71.5 ± 12.2
	5	90.8 ± 14.1	127.1 ± 16.8	35.8 ± 7.3	21.8 ± 4.3	18.7 ± 3.6	57.8 ± 13.6
	6	79.4 ± 10.8	122.8 ± 14.4	23.9 ± 3.2	33.6 ± 4.8	18.5 ± 2.6	69.1 ± 11.8
	7	84.9 ± 13.4	**134.3 ± 23.0**	**49.4 ± 15.1**	**41.4 ± 9.6**	18.0 ± 3.1	57.9 ± 15.6
NJ	0	21.4 ± 5.4	83.7 ± 28.6	30.1 ± 12.1	32.3 ± 7.6	18.2 ± 9.3	78.6 ± 14.6
	1	22.5 ± 5.6	84.4 ± 29.4	80.5 ± 29.5	36.5 ± 6.1	13.7 ± 9.1	78.3 ± 12.2
	2	22.6 ± 5.5	78.8 ± 31.8	42.0 ± 9.3	55.9 ± 8.4	13.6 ± 8.1	69.5 ± 15.3
	3	20.9 ± 5.5	185.2 ± 37.4	36.1 ± 10.9	33.8 ± 7.7	25.7 ± 4.9	68.4 ± 9.6
	4	**24.0 ± 5.8**	77.9 ± 31.9	**106.4 ± 46.6**	**71.4 ± 18.3**	9.8 ± 6.2	69.8 ± 12.7
	5	20.4 ± 4.3	**187.8 ± 36.0**	74.0 ± 18.6	36.5 ± 5.7	24.4 ± 4.6	73.6 ± 8.3
	6	20.9 ± 5.6	178.5 ± 31.4	41.1 ± 9.7	54.7 ± 10.4	22.2 ± 5.1	62.7 ± 11.7
	7	21.3 ± 5.4	185.8 ± 42.2	87.9 ± 30.2	65.8 ± 16.1	20.6 ± 5.2	64.8 ± 10.9
WH	0	55.8 ± 10.7	85.4 ± 35.9	34.2 ± 13.3	30.6 ± 7.7	19.6 ± 8.3	82.9 ± 12.0
	1	60.6 ± 13.0	74.8 ± 36.5	95.7 ± 39.8	38.3 ± 6.2	12.2 ± 7.8	83.3 ± 9.3
	2	59.8 ± 11.2	85.0 ± 36.5	50.4 ± 9.0	54.1 ± 9.3	14.6 ± 7.4	79.7 ± 10.3
	3	53.9 ± 10.3	178.7 ± 24.4	40.2 ± 10.8	33.1 ± 7.0	27.4 ± 3.9	74.7 ± 8.5
	4	**64.4 ± 13.5**	73.5 ± 35.0	**125.1 ± 58.8**	**72.6 ± 19.0**	9.0 ± 6.0	78.7 ± 9.5
	5	52.3 ± 8.3	186.3 ± 31.2	79.7 ± 21.0	38.3 ± 6.0	25.1 ± 3.3	76.2 ± 7.1
	6	54.3 ± 9.5	185.8 ± 31.2	49.5 ± 9.0	58.3 ± 9.3	22.4 ± 4.5	73.5 ± 9.1
	7	53.6 ± 9.4	**193.5 ± 33.4**	93.9 ± 29.0	68.1 ± 16.9	21.5 ± 4.5	77.2 ± 6.8
WLMQ	0	22.0 ± 5.8	61.1 ± 18.9	29.2 ± 9.8	35.2 ± 8.6	10.1 ± 9.0	58.3 ± 20.4
	1	21.5 ± 5.6	43.6 ± 23.2	74.4 ± 25.1	41.6 ± 7.9	2.4 ± 9.4	70.3 ± 22.0
	2	21.7 ± 5.3	60.9 ± 18.3	37.5 ± 8.1	60.1 ± 9.9	7.9 ± 11.0	54.5 ± 18.4
	3	21.1 ± 6.3	118.1 ± 22.1	29.6 ± 8.7	37.9 ± 8.4	22.6 ± 4.0	41.9 ± 13.1
	4	21.7 ± 6.0	37.2 ± 21.1	145.3 ± 76.2	**77.2 ± 18.2**	−3.6 ± 9.4	70.5 ± 16.7
	5	**24.0 ± 16.3**	113.6 ± 20.2	65.8 ± 36.4	42.0 ± 6.8	22.6 ± 4.3	38.5 ± 11.3
	6	18.7 ± 5.3	**125.6 ± 25.8**	37.8 ± 7.1	58.2 ± 5.8	23.1 ± 4.0	34.5 ± 9.0
	7	19.0 ± 5.8	120.6 ± 24.8	**66.8 ± 14.2**	65.5 ± 12.6	21.5 ± 5.2	34.3 ± 8.6
XA	0	103.1 ± 25.6	66.9 ± 27.0	34.9 ± 12.2	32.0 ± 7.3	14.5 ± 8.4	70.2 ± 19.6
	1	111.2 ± 25.2	57.6 ± 27.5	84.9 ± 30.6	36.7 ± 6.4	9.1 ± 8.0	69.8 ± 16.9
	2	110.8 ± 25.6	74.0 ± 27.6	44.4 ± 9.5	53.2 ± 9.6	12.1 ± 8.1	59.8 ± 16.7
	3	94.8 ± 19.3	162.0 ± 32.4	36.7 ± 11.1	33.6 ± 6.6	26.1 ± 3.6	61.4 ± 10.6
	4	**112.5 ± 25.8**	54.4 ± 27.3	**139.4 ± 86.2**	**65.7 ± 15.6**	6.5 ± 6.9	64.9 ± 15.0
	5	87.9 ± 20.4	166.8 ± 31.1	79.6 ± 23.3	35.8 ± 5.8	25.6 ± 3.8	62.6 ± 11.5
	6	92.7 ± 17.9	**174.0 ± 40.8**	42.4 ± 9.8	54.3 ± 8.5	24.1 ± 3.9	58.2 ± 10.2
	7	94.4 ± 23.8	156.8 ± 28.4	100.9 ± 47.4	**65.7 ± 14.6**	20.7 ± 4.5	60.5 ± 12.6

## References

[1] Chan C.K., Yao X. (2008). Air Pollution in Mega Cities in China. Atmos. Environ..

[2] Tao Y., Huang W., Huang X., Zhong L., Lu S.-E., Li Y., Dai L., Zhang Y., Zhu T. (2012). Estimated Acute Effects of Ambient Ozone and Nitrogen Dioxide on Mortality in the Pearl River Delta of Southern China. Environ. Health Perspect..

[3] Khaefi M., Goudarzi G., Yari A., Geravandi S., Dobaradaran S., Idani E., Javanmardi P., Youesfi F., Hashemzadeh B., Shahriari A. (2016). An Association between Ambient Pollutants and Hospital Admitted Respiratory Cases in Ahvaz, Iran. Fresenius Environ. Bull..

[4] Dastoorpoor M., Goudarzi G., Khanjani N., Idani E., Aghababaeian H., Bahrampour A. (2018). Lag Time Structure of Cardiovascular Deaths Attributed to Ambient Air Pollutants in Ahvaz, Iran, 2008–2015. Int. J. Occup. Med. Environ. Health.

[5] Javanmardi P., Morovati P., Farhadi M., Geravandi S., Khaniabadi Y.O., Angali K.A., Taiwo A.M., Sicard P., Goudarzi G., Valipour A. (2018). Monitoring the Impact of Ambient Ozone on Human Health Using Time Series Analysis and Air Quality Model Approaches. Fresenius Environ. Bull..

[6] WHO (2021). New WHO Global Air Quality Guidelines Aim to Save Millions of Lives from Air Pollution. https://www.who.int/news/item/22-09-2021-new-who-global-air-quality-guidelines-aim-to-save-millions-of-lives-from-air-pollution.

[7] Ministry of Ecology and Environment of the People’s Republic of China (2015). China Environmental Status Bulletin. http://www.gov.cn/xinwen/2016-06/02/5078966/files/9ab14b4ce3294d5ab212bc83d3d31b7b.pdf.

[8] Bell M.L., Kim J.Y., Dominici F. (2007). Potential Confounding of Particulate Matter on the Short-Term Association between Ozone and Mortality in Multisite Time-Series Studies. Environ. Health Perspect..

[9] Rojas-Martinez R., Perez-Padilla R., Olaiz-Fernandez G., Mendoza-Alvarado L., Moreno-Macias H., Fortoul T., McDonnell W., Loomis D., Romieu I. (2007). Lung Function Growth in Children with Long-Term Exposure to Air Pollutants in Mexico City. Am. J. Respir. Crit. Care Med..

[10] Dominici F., Peng R.D., Barr C.D., Bell M.L. (2010). Protecting Human Health from Air Pollution: Shifting from a Single-Pollutant to a Multi-Pollutant Approach. Epidemiol. Camb. Mass.

[11] Cao J., Xu H., Xu Q., Chen B., Kan H. (2012). Fine Particulate Matter Constituents and Cardiopulmonary Mortality in a Heavily Polluted Chinese City. Environ. Health Perspect..

[12] Chen R., Yin P., Meng X., Liu C., Wang L., Xu X., Ross J.A., Tse L.A., Zhao Z., Kan H. (2017). Fine Particulate Air Pollution and Daily Mortality. A Nationwide Analysis in 272 Chinese Cities. Am. J. Respir. Crit. Care Med..

[13] Atkinson R.W., Yu D., Armstrong B.G., Pattenden S., Wilkinson P., Doherty R.M., Heal M.R., Anderson H.R. (2012). Concentration—Response Function for Ozone and Daily Mortality: Results from Five Urban and Five Rural, U.K. Populations. Environ. Health Perspect..

[14] Yin P., Chen R., Wang L., Meng X., Liu C., Niu Y., Lin Z., Liu Y., Liu J., Qi J. (2017). Ambient Ozone Pollution and Daily Mortality: A Nationwide Study in 272 Chinese Cities. Environ. Health Perspect..

[15] Duan Y., Liao Y., Li H., Yan S., Zhao Z., Yu S., Fu Y., Wang Z., Yin P., Cheng J. (2019). Effect of Changes in Season and Temperature on Cardiovascular Mortality Associated with Nitrogen Dioxide Air Pollution in Shenzhen, China. Sci. Total Environ..

[16] Mauderly J.L., Samet J.M. (2009). Is There Evidence for Synergy Among Air Pollutants in Causing Health Effects?. Environ. Health Perspect..

[17] WHO (2018). WHO Expert Consultation: Available Evidence for the Future Update of the WHO Global Air Quality Guidelines (AQGs).

[18] Stieb D.M., Burnett R.T., Smith-Doiron M., Brion O., Shin H.H., Economou V. (2008). A New Multipollutant, No-Threshold Air Quality Health Index Based on Short-Term Associations Observed in Daily Time-Series Analyses. J. Air Waste Manag. Assoc..

[19] Sicard P., Lesne O., Alexandre N., Mangin A., Collomp R. (2011). Air Quality Trends and Potential Health Effects—Development of an Aggregate Risk Index. Atmos. Environ..

[20] Olstrup H., Johansson C., Forsberg B., Tornevi A., Ekebom A., Meister K. (2019). A Multi-Pollutant Air Quality Health Index (AQHI) Based on Short-Term Respiratory Effects in Stockholm, Sweden. Int. J. Environ. Res. Public. Health.

[21] Bobb J.F., Valeri L., Claus Henn B., Christiani D.C., Wright R.O., Mazumdar M., Godleski J.J., Coull B.A. (2015). Bayesian Kernel Machine Regression for Estimating the Health Effects of Multi-Pollutant Mixtures. Biostatistics.

[22] Godzinski A., Suarez Castillo M. (2021). Disentangling the Effects of Air Pollutants with Many Instruments. J. Environ. Econ. Manag..

[23] The Ministry of Housing and Urban-Rural Development of the People’s Republic of China (2019). China Urban Construction Statistical Yearbook: 2019.

[24] Bell M.L. (2004). Ozone and Short-Term Mortality in 95 US Urban Communities, 1987–2000. JAMA.

[25] Romieu I., Gouveia N., Cifuentes L.A., de Leon A.P., Junger W., Vera J., Strappa V., Hurtado-Díaz M., Miranda-Soberanis V., Rojas-Bracho L. (2012). Multicity Study of Air Pollution and Mortality in Latin America (the ESCALA Study). Res. Rep. Health Eff. Inst..

[26] Thurston G.D., Ito K. (2001). Epidemiological Studies of Acute Ozone Exposures and Mortality. J. Expo. Sci. Environ. Epidemiol..

[27] Ito K., Thurston G.D., Nádas A., Lippmann M. (2001). Monitor-to-Monitor Temporal Correlation of Air Pollution and Weather Variables in the North-Central US. J. Expo. Sci. Environ. Epidemiol..

[28] Wong C.-M., Vichit-Vadakan N., Kan H., Qian Z. (2008). Public Health and Air Pollution in Asia (PAPA): A Multicity Study of Short-Term Effects of Air Pollution on Mortality. Environ. Health Perspect..

[29] Lin H., Tao J., Du Y., Liu T., Qian Z., Tian L., Di Q., Rutherford S., Guo L., Zeng W. (2016). Particle Size and Chemical Constituents of Ambient Particulate Pollution Associated with Cardiovascular Mortality in Guangzhou, China. Environ. Pollut..

[30] CAA (2020). China Air 2020: Air Pollution Prevention and Control Progress in Chinese Cities.

[31] Stafoggia M., Breitner S., Hampel R., Basagaña X. (2017). Statistical Approaches to Address Multi-Pollutant Mixtures and Multiple Exposures: The State of the Science. Curr. Environ. Health Rep..

[32] Sun Z.B., An X.Q., Cui M.M., Tao Y., Ma X.H., Ye C. (2016). The effect of PM2.5 and PM10 on cardiovascular and cerebrovascular diseases admission visitors in Beijing areas during dust weather, non-dust weather and haze pollution. China Environ. Sci..

[33] Zhang Y., Zhang X., Fan X., Ni C., Sun Z., Wang S., Fan J., Zheng C. (2020). Modifying Effects of Temperature on Human Mortality Related to Black Carbon Particulates in Beijing, China. Atmos. Environ..

[34] Joundi R.A., Patten S.B., Williams J.V.A., Smith E.E. (2021). Association Between Excess Leisure Sedentary Time and Risk of Stroke in Young Individuals. Stroke.

[35] Ostro B., Broadwin R., Green S., Feng W.-Y., Lipsett M. (2006). Fine Particulate Air Pollution and Mortality in Nine California Counties: Results from CALFINE. Environ. Health Perspect..

[36] Samet J.M., Katsouyanni K., Team P.I. (2006). for the A. Air Pollution and Health: A Combined European and North American Approach (APHENA). Epidemiology.

[37] Papathomas M., Molitor J., Richardson S., Riboli E., Vineis P. (2011). Examining the Joint Effect of Multiple Risk Factors Using Exposure Risk Profiles: Lung Cancer in Nonsmokers. Environ. Health Perspect..

[38] Wesson K., Fann N., Morris M., Fox T., Hubbell B. (2010). A Multi-Pollutant, Risk-Based Approach to Air Quality Management: Case Study for Detroit. Atmos. Pollut. Res..

[39] Huang W.-Z., He W.-Y., Knibbs L.D., Jalaludin B., Guo Y.-M., Morawska L., Heinrich J., Chen D.-H., Yu Y.-J., Zeng X.-W. (2022). Improved Morbidity-Based Air Quality Health Index Development Using Bayesian Multi-Pollutant Weighted Model. Environ. Res..

[40] Zhu J., Chen L., Liao H. (2022). Multi-Pollutant Air Pollution and Associated Health Risks in China from 2014 to 2020. Atmos. Environ..

[41] Traini E., Huss A., Portengen L., Rookus M., Verschuren W.M.M., Vermeulen R.C.H., Bellavia A. (2022). A Multipollutant Approach to Estimating Causal Effects of Air Pollution Mixtures on Overall Mortality in a Large, Prospective Cohort. Epidemiol. Camb. Mass.

[42] IQ Air (2021). World Air Quality Report: Region and City PM2.5 Ranking.

[43] Yan M., Wilson A., Bell M.L., Peng R.D., Sun Q., Pu W., Yin X., Li T., Anderson G.B. (2019). The Shape of the Concentration—Response Association between Fine Particulate Matter Pollution and Human Mortality in Beijing, China, and Its Implications for Health Impact Assessment. Environ. Health Perspect..

[44] Wong C.M., Ma S., Hedley A.J., Lam T.H. (2001). Effect of Air Pollution on Daily Mortality in Hong Kong. Environ. Health Perspect..

[45] Burkart K., Canário P., Breitner S., Schneider A., Scherber K., Andrade H., Alcoforado M.J., Endlicher W. (2013). Interactive Short-Term Effects of Equivalent Temperature and Air Pollution on Human Mortality in Berlin and Lisbon. Environ. Pollut..

[46] Stafoggia M., Schwartz J., Forastiere F., Perucci C.A. (2008). Does Temperature Modify the Association between Air Pollution and Mortality? A Multicity Case-Crossover Analysis in Italy. Am. J. Epidemiol..

[47] Meng X., Zhang Y., Zhao Z., Duan X., Xu X., Kan H. (2012). Temperature Modifies the Acute Effect of Particulate Air Pollution on Mortality in Eight Chinese Cities. Sci. Total Environ..

[48] Li G., Zhou M., Cai Y., Zhang Y., Pan X. (2011). Does Temperature Enhance Acute Mortality Effects of Ambient Particle Pollution in Tianjin City, China. Sci. Total Environ..

[49] Qian Z., He Q., Lin H.-M., Kong L., Bentley C.M., Liu W., Zhou D. (2008). High Temperatures Enhanced Acute Mortality Effects of Ambient Particle Pollution in the “Oven” City of Wuhan, China. Environ. Health Perspect..

[50] Li Y., Ma Z., Zheng C., Shang Y. (2015). Ambient Temperature Enhanced Acute Cardiovascular-Respiratory Mortality Effects of PM2.5 in Beijing, China. Int. J. Biometeorol..

